# An Asian Perspective: The dataset for validation of Teachers’ Information and Communication Technology Access (TICTA)

**DOI:** 10.1016/j.dib.2020.105592

**Published:** 2020-04-22

**Authors:** Lantip Diat Prasojo, Akhmad Habibi, Sutrisna Wibawa, Prosmala Hadisaputra, Amirul Mukminin, Muhaimin, Mohd Faiz Mohd Yaakob

**Affiliations:** a Universitas Negeri Yogyakarta; b LPDP Indonesia; c Universitas Jambi; d Universiti Utara Malaysia

**Keywords:** TICTA, Survey, K-12 school, Teachers, Educational technology

## Abstract

This dataset presents the validation process of a survey of factors affecting Indonesian K-12 school teachers’ Teachers’ Information and Communication Technology Access (TICTA). An initial instrument was developed through the adaptation of instruments from previous studies. Afterward, it was piloted to 120 teachers and tested for its reliability. For the main data collection, the instrument was distributed online and responded by 2775 Indonesian K-12 school teachers. The main data analysis was conducted for the measurement model using four assessments; reflective indicator loadings, internal consistency reliability, convergent, and discriminant validity. The Partial Least Square Structural Equation Model (PLS-SEM) was utilized for the analysis. The dataset is beneficial for educational regulators in providing appropriate access to ICT in K-12 education and for educational researchers for future research on technology access in teaching.

Specifications table

**Table:** 

Subject	Education
Specific subject area	Educational technology
Type of data	Table
	Figure
How data were acquired	Face and content validity, survey, and PLS-SEM Measurement model
Data format	Raw
	Analyzed
	Filtered
Parameters for data collection	The instrument includes demographic information, Endogenous Motivational Access, Exogenous Motivational Access, Operational Skills Access, Informational Skills Access, Strategic Skills Access, General Usage Access, Instructional Usage Access,
Description of data collection	The instrument was adapted from previous studies, translated and validated through content validity and pilot study. The analysis of the data was done using PLS-SEM, measurement model.
Data source location	Region: Yogyakarta, Jambi, East Java, and Bangka Belitung
	Country: Indonesia
	Latitude and longitude (and GPS coordinates) for collected samples/data:0.7893° S, 113.9213° E
Data accessibility	On a public repository:
	Repository name: Mandeley Data
	Data identification number: 10.17632/gmhfnzfj9w.2
	Direct URL to data: http://dx.doi.org/10.17632/gmhfnzfj9w.2

## Value of the data

• The dataset presents a validation process of a survey of factors predicting General Usage Access and Instructional Usage Access of Information and Communication Technology (ICT) from Indonesian K-12 teachers.
• The dataset is beneficial for educational regulators in providing appropriate access of ICT in K-12 education
• Access to this dataset may contribute to educational researchers for future research on TICTA

## 1 Data Description

Data of this survey study include eight variables with 29 items adapted from previous studies [1], [2], [3], [4]. .. Six independent variables are Endogenous Motivational Access or EnMA (3 items), Exogenous Motivation Access or ExMA (4 items), Strategic Skill Access or SSA (4 items), Operational Skills Access or OSA (4 items) and Informational Skills Access or ISA (5 items). Meanwhile, two dependent variables are General Use Access or GUA (4 items) and Instructional Use Access or IUA (5 items). the survey instrument is accessible online at https://forms.gle/AGWT1U778vqrPPHq6.

## 2 Experimental design, materials, and methods

The main data collection was conducted through an online survey through stratified random sampling. After the data conversion, the normality assessment was done by calculating Skewness and Kurtosis [6]. Skewness and Kurtosis values need to be in a range of -2 to +2 [source]. All Skewness and Kurtosis values are within the recommended range values (Table 1). All items’ value of Skewness and Kurtosis meet the threshold. Mean and Standard Deviation (SD) are also reported in this early stage.

**Table 1:** Mean, SD, Skewness, and Kurtosis.

Items	Mean	SD	Skewness		Kurtosis	
	Statistic	Statistic	Statistic	SE	Statistic	SE
EnMA1	4.360	0.687	-1.020	0.046	1.721	0.093
EnMA2	4.497	0.638	-1.313	0.046	2.860	0.093
EnMA3	4.442	0.668	-1.207	0.046	2.359	0.093
ExMA1	4.325	0.706	-0.979	0.046	1.500	0.093
ExMA2	4.061	0.814	-0.753	0.046	0.737	0.093
ExMA3	3.722	1.065	-0.780	0.046	0.161	0.093
ExMA4	3.854	1.004	-0.827	0.046	0.352	0.093
OSA1	4.367	0.701	-0.987	0.046	1.166	0.093
OSA2	4.257	0.753	-0.827	0.046	0.570	0.093
OSA3	4.381	0.721	-1.104	0.046	1.372	0.093
OSA4	4.355	0.767	-1.166	0.046	1.354	0.093
ISA1	3.986	0.844	-0.563	0.046	0.035	0.093
ISA2	4.119	0.754	-0.598	0.046	0.248	0.093
ISA3	4.008	0.765	-0.439	0.046	0.015	0.093
ISA4	4.142	0.791	-0.737	0.046	0.376	0.093
ISA5	4.161	0.761	-0.690	0.046	0.380	0.093
SSA1	4.174	0.704	-0.592	0.046	0.412	0.093
SSA2	4.444	0.635	-0.951	0.046	1.119	0.093
SSA3	3.977	0.811	-0.493	0.046	0.006	0.093
SSA4	4.078	0.757	-0.534	0.046	0.150	0.093
GUA1	4.333	0.700	-0.912	0.046	0.970	0.093
GUA2	3.769	1.073	-0.689	0.046	-0.144	0.093
GUA3	3.966	0.907	-0.697	0.046	0.189	0.093
GUA4	3.907	1.002	-0.797	0.046	0.173	0.093
IUA1	3.688	0.946	-0.554	0.046	0.077	0.093
IUA2	4.019	0.797	-0.721	0.046	0.773	0.093
IUA3	3.774	0.888	-0.534	0.046	0.205	0.093
IUA4	3.812	0.863	-0.589	0.046	0.414	0.093
IUA5	4.028	0.835	-0.728	0.046	0.468	0.093

**Fig. 1:**
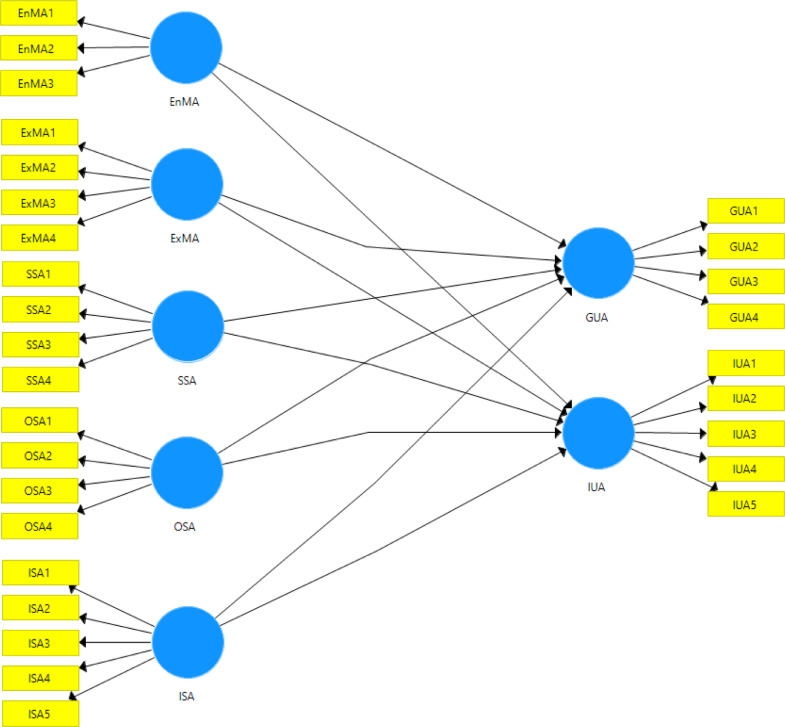
Proposed model.

After data screening and cleaning, the analysis of the data was conducted to 2775 measurable responses (Table 2). For the reflective measurement model, we employed Partial Least Square- Structural Equation Modeling (PLS-SEM) to measure four measurements of the proposed model (Fig. 1), namely the reflective indicator loadings, internal consistency reliability, convergent, and discriminant validity [5]. The indicator loading should be 0.708 or higher. The internal consistency reliability was reported using Cronbach's alpha (> 0.700) and Composite Reliability (CR) with the range of .700 to .900 for the threshold values [5]. We reported the convergent validity through Average Variance Extracted (AVE) values (≥ 0.500). Table 3 provides the information of the four measurements. All loading values meet the threshold (0.708-.0922). The Cronbach's alpha value is between 0.733 and 0.883. AVE ranges from 0.548 to 0.803.

**Table 2:** Demographic information.

Demographic	N	%

***Province***		
Jambi	658	23.71
Yogyakarta	1197	43.14
East Java	302	10.88
Bangka Belitung	618	22.27
***Gender***		
Female	1656	59.68
Male	1119	40.32
***Experience***		
< 5 years	526	18.95
5-10 years	1724	62.13
> 10 years	525	18.92
***Level of school***		
Elementary school level	387	13.95
Junior high school level	836	30.13
Senior high school level	1552	55.93
***Access to ICT***		
**Computer**		
Yes	2261	81.48
No	514	18.52
**Laptop**		
Yes	2715	97.84
No	60	2.16
**Smartphone**		
Yes	2674	96.36
No	101	3.64
**Tablet**		
Yes	645	23.24
No	2130	76.76

**Table 3:** Item, Loading, Cronbach's Alpha, CR, and AVE.

Variable	Item	Loading	Cronbach's Alpha	CR	AVE
EnMA	EnMA1	0.846	0.852	0.910	0.772
	EnMA2	0.898			
	EnMA3	0.890			
ExMA	ExMA2	0.745	0.733	0.829	0.548
	ExMA3	0.708			
	ExMA4	0.745			
GUA	GUA1	0.811	0.811	0.875	0.637
	GUA2	0.753			
	GUA3	0.808			
	GUA4	0.819			
ISA	ISA1	0.849	0.876	0.924	0.803
	ISA4	0.915			
	ISA5	0.922			
IUA	IUA1	0.825	0.883	0.920	0.741
	IUA2	0.892			
	IUA4	0.880			
	IUA5	0.844			
OSA	OSA1	0.875	0.851	0.910	0.771
	OSA2	0.886			
	OSA4	0.872			
SSA	SSA1	0.872	0.881	0.918	0.737
	SSA2	0.828			
	SSA3	0.846			
	SSA4	0.888			

**Fig. 2:**
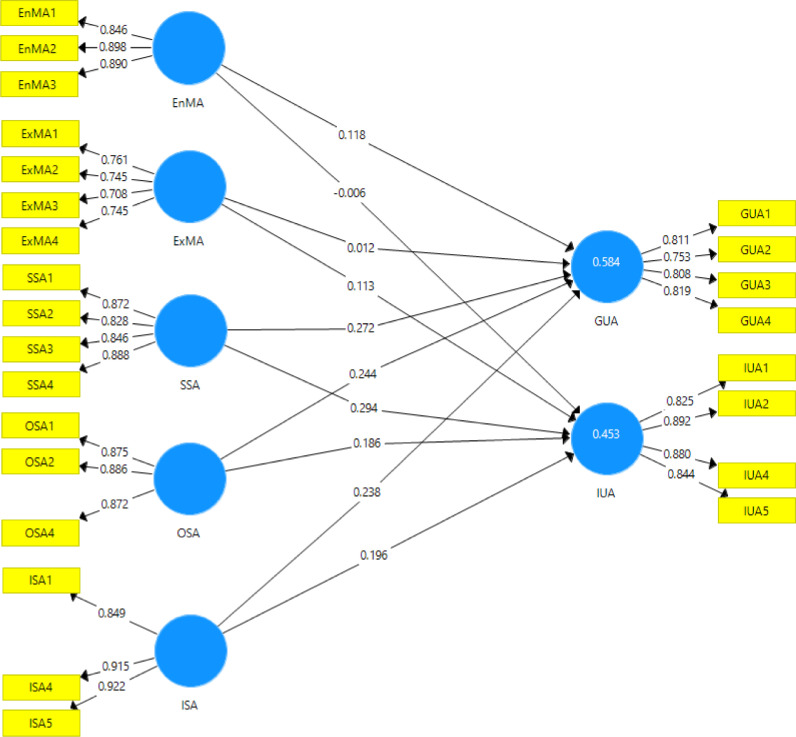
Final model.

Besides, discriminant validity was reported using Heterotrait-Monotrait Ratio (HTMT) that the value should be less than 0.850 [5]. All HTMT correlation values are less than 0.850 of all variables (Table 4). Four items were dropped due to low loading values (ISA 2, 3; ExMA 1, and OSA 3). The final model after the measurement model consists of eight variables and 25 items (Fig. 2). The loading values and path coefficients of the model can be seen in Fig. 2. SmartPLS 3.0 program for PLS_SEM was applied to compute and estimate the model.

**Table 4:** HTMT.

	EnMA	ExMA	GUA	ISA	IUA	OSA
ExMA	0.675					
GUA	0.654	0.553				
ISA	0.589	0.527	0.791			
IUA	0.513	0.538	0.810	0.665		
OSA	0.675	0.560	0.798	0.816	0.660	
SSA	0.721	0.680	0.804	0.824	0.704	0.792
